# Amnion epithelial cells are an effective source of factor H and prevent kidney complement deposition in factor H-deficient mice

**DOI:** 10.1186/s13287-021-02386-7

**Published:** 2021-06-10

**Authors:** Federica Casiraghi, Pamela Yossenaidy Rodriguez Ordonez, Nadia Azzollini, Marta Todeschini, Daniela Rottoli, Roberta Donadelli, Roberto Gramignoli, Ariela Benigni, Marina Noris, Giuseppe Remuzzi

**Affiliations:** 1 Istituto di Ricerche Farmacologiche Mario Negri IRCCS, Via GB Camozzi 3, 24020 Ranica, Bergamo Italy; 2 Department of Laboratory Medicine, Division of Pathology, Karolinska Institutet, Stockholm, Sweden

**Keywords:** Human amnion epithelial cells, Complement factor H, Complement alternative pathway, Renal glomeruli, Complement deposition

## Abstract

Complement factor H (FH) is the main plasma regulator of the alternative pathway of complement. Genetic and acquired abnormalities in FH cause uncontrolled complement activation amplifying, with the consequent accumulation of complement components on the renal glomeruli. This leads to conditions such as C3 glomerulopathy (C3G) and atypical hemolytic uremic syndrome (aHUS). There is no effective therapy for these diseases. Half of the patients progress to end-stage renal disease and the condition recurs frequently in transplanted kidneys. Combined liver/kidney transplantation is a valid option for these patients, but the risks of the procedure and donor organ shortages hamper its clinical application. Therefore, there is an urgent need for alternative strategies for providing a normal FH supply. Human amnion epithelial cells (hAEC) have stem cell characteristics, including the capability to differentiate into hepatocyte-like cells in vivo.

Here, we administered hAEC into the livers of newborn *Cfh*^*−/−*^ mice, which spontaneously developed glomerular complement deposition and renal lesions resembling human C3G. hAEC engrafted at low levels in the livers of *Cfh*^*−/−*^ mice and produced sufficient human FH to prevent complement activation and glomerular C3 and C9 deposition. However, long-term engraftment was not achieved, and eventually hAEC elicited a humoral immune response in immunocompetent *Cfh*^*−/−*^ mice.

hAEC cell therapy could be a valuable therapeutic option for patients undergoing kidney transplantation in whom post-transplant immunosuppression may protect allogeneic hAEC from rejection, while allogeneic cells provide normal FH to prevent disease recurrence.

**Supplementary Information:**

The online version contains supplementary material available at 10.1186/s13287-021-02386-7.

## Significance statement

Patients with genetic deficiency or abnormalities in complement factor H (FH)—the main regulator of the alternative pathway of complement—develop renal disease that progresses to end-stage renal disease. Here, we administered human amnion epithelial cell (hAEC) to FH-deficient mice to assess whether these stem cells could be an effective source of normal FH. We found that hAEC engrafted into the murine liver and produced FH protein at levels capable of preventing complement activation in the kidney. Therefore, we suggest that hAEC can be an effective cellular strategy for preventing disease recurrence in patients with FH deficiency undergoing kidney transplantation.

## Introduction

The complement system belongs to the innate immune system and represents the first line of defense against pathogens [1]. Complement activation occurs via three pathways: the classical pathway, activated by surface-bound immunoglobulins; the lectin pathway, activated by distinct carbohydrate moieties on the pathogen surface; and the alternative pathway, which is continuously activated via the spontaneous turn-over of complement C3. All three pathways converge in the sequential formation of C3 and C5 convertases on the cell surface, culminating in the assembly of the cytolytic complex C5b-9 [1, 2].

The harmful effect of complement activation via the alternative pathway on host cells is tightly controlled by several regulators, of which factor H (FH) is the major regulator, both in the fluid-phase and on the cell surface [3]. Genetic deficiencies or abnormalities of FH lead to uncontrolled C3 activation and the accumulation of C3 and C5 activation products within the renal glomerulus [4] and cause the rare renal diseases C3 glomerulopathy (C3G) and atypical hemolytic uremic syndrome (aHUS) [5].

The prognosis for C3G and aHUS is poor; about 50% of patients develop end-stage renal disease, and both diseases recur in the donor kidney in 50–80% of transplant recipients [6]. In most aHUS patients, the anti-C5 antibody eculizumab induces disease remission and prevents recurrences. However, it is only partially effective in C3G [7].

Since FH is synthesized mostly by the liver, combined liver and kidney transplantations have been performed successfully in patients with FH gene mutations, in whom normal FH produced by the donor liver prevented disease recurrence in the kidney graft [8]. However, the invasiveness and risks of the procedure, as well as organ shortages, have severely limited its clinical application. Replacement therapy with exogenous FH would be the most logical therapeutic solution. However, the size, complexity, and short in vivo half-life of plasma-derived, recombinant full- or mini-FH protein have hampered its biopharmaceutical development [3, 9–12].

An alternative strategy could be cellular therapy, with allogeneic healthy cells as a source of wild-type FH. Human amnion epithelial cells (hAEC) have stem cell characteristics and the ability to differentiate into functional hepatocytes in vivo [13, 14]. In preclinical studies, hAEC have been used successfully to treat monogenic liver diseases, such as intermediate maple syrup urine disease (iMSUD) [15, 16] and Hurler syndrome [17], where transplanted hAEC compensated for the missing activity of branched chain α-keto acid dehydrogenase (BCKDH) and α-L-iduronidase (IDUA) enzymes, respectively. Since hepatocytes are the main source of FH [18], here we tested the therapeutic efficacy of intra-liver hAEC therapy in controlling complement activation in *Cfh*^*−/−*^ mice that spontaneously develop a renal disease resembling C3G [19].

## Materials and methods

Detailed methods are shown in the Supplemental Information online.

### Experimental design

#### hAEC transplantation

*Cfh*^*−/−*^ mice [19] (kindly gifted by Dr Matthew Pickering at Imperial College in London) were given hAEC by direct hepatic injection at 10 days and then via the portal vein at 30 days of age (1 × 10^6^ cells each injection) or PBS and euthanized either 10 or 40 days after the last hAEC/PBS injection (*n* = 3–4 mice/group) (Supplemental Figure S1).

## Results

### hAEC engrafted at a low level into the liver of *Cfh*^*−/−*^ mice and express FH mRNA

**Fig. 1:**
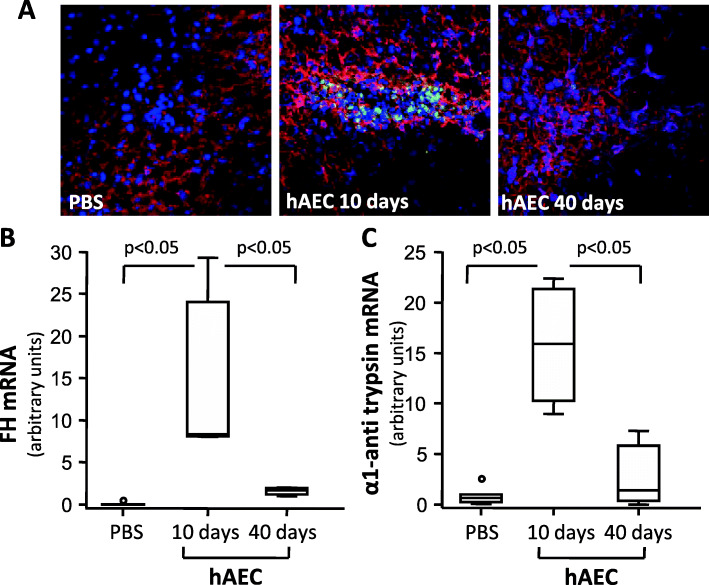
*hAEC engrafted in the liver and expressed FH and α1 anti-trypsin mRNA.* **A** Representative images of HNA^+^ (green) and DAPI^+^ (blu) cells in livers from *Cfh*^*−/−*^ mice given either PBS or hAEC injection and euthanized 10 or 40 days later. No HNA-positive hAEC were detected in the livers of mice given PBS. The liver sections were counterstained for hepatocyte antigen expression (red staining). Original magnification × 400. mRNA expression of human FH (**B**) and anti-trypsin (**C**) in the livers of *Cfh*^*−/−*^ mice given either PBS or hAEC injection and euthanized 10 or 40 days after. Horizontal lines indicated statistically significant differences (*P* < 0.05) between groups

Immunofluorescence analysis of hepatic tissues of hAEC-injected *Cfh*^*−/−*^ mice revealed the presence of HNA-positive cells both 10 and 40 days post-cell injection (Fig. 1A). Engrafted hAEC accounted for about 3% of cells in the liver (2.86 ± 0.85 % HNA^+^ cells/nuclei) at 10 days, and decreased to the very low level of 0.15% (0.15±0.04) at day 40 post-injection.

To verify whether engrafted hAEC differentiated into FH-producing hepatocyte-like cells, we evaluated mRNA expression for human FH in livers from hAEC-injected mice. We also evaluated mRNA expression of α1 anti-trypsin (α1AT) as one of the major mature liver metabolic enzymes [20].

Undifferentiated hAEC expressed very low levels of FH and α1AT mRNA compared to the human liver (2.3 × 10^−5^ and 2.5 × 10^−3^ considering mRNA from human liver=1, respectively). Ten days after cell injection, the livers of hAEC-treated *Cfh*^*−/−*^ mice exhibited significantly higher mRNA levels of human FH and α1AT than PBS-treated *Cfh*^*−/−*^ mice. In livers from hAEC-treated mice analyzed 40 days after cell injection, mRNA levels of FH and α1AT decreased significantly compared to mice euthanized 10 days after hAEC injection, (Fig. 1B, `C). Serum levels of human FH in hAEC injected mice were below the detection limit of the ELISA assay (3 ng/mL) at both time points.

### hAEC treatment increased C3 serum levels and decreased complement glomerular deposits in *Cfh*^*−/−*^ mice

**Fig. 2:**
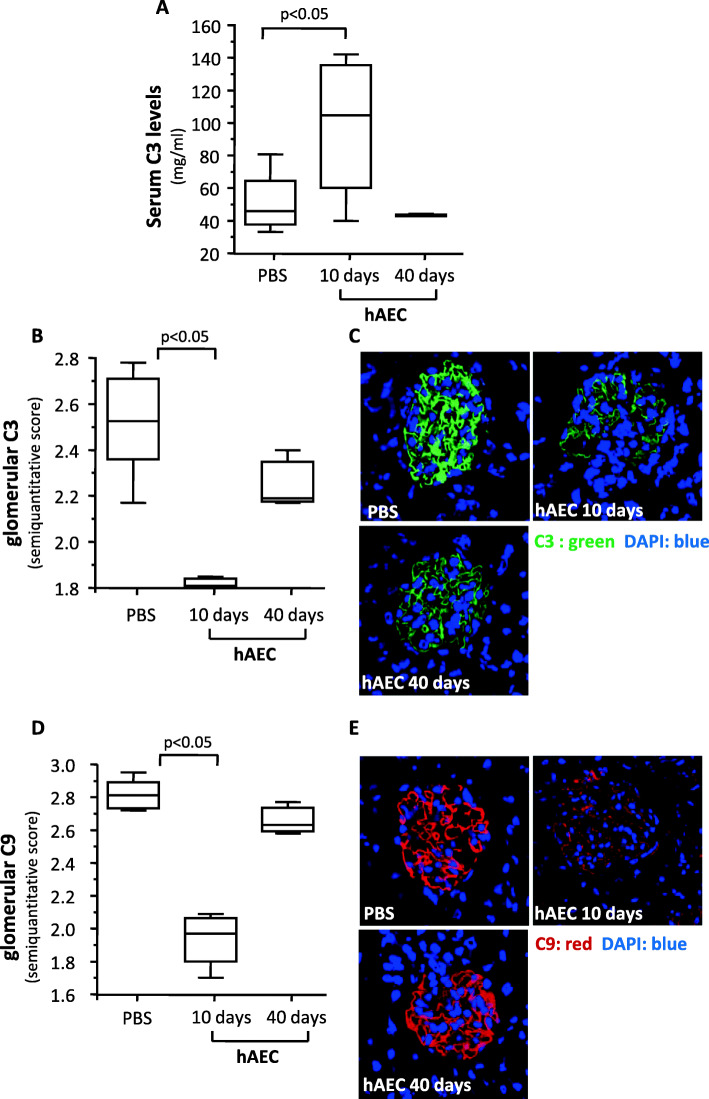
*hAEC reduced serum C3 levels and glomerular C3 and C9 staining in Cfh*^*−/−*^ *mice.* Serum C3 level (**A**) and semiquantitative scores of glomerular C3 (**B**) and C9 (**D**) staining in *Cfh*^*−/−*^ mice given PBS or hAEC injections and sacrificed either 10 or 40 days later. Green horizontal lines indicated statistically significant differences (*P* < 0.05) between groups. Mice given PBS as controls for hAEC and sacrificed 10 or 40 days after injection exhibited non-significant differences in all the considered parameters (Supplemental Figure S2) and were therefore pooled in a single control group (*n* = 8). Panels **C** and **E** show representative images of C3 (green) and C9 (red) deposits in kidneys counterstained with DAPI (blue) of *Cfh*^*−/−*^ from the indicated experimental group. Original magnification × 630

*Cfh*^*−/−*^ mice develop uncontrolled activation of the alternative pathway, leading to marked accumulation of C3 and C9 in glomeruli and low levels of circulating C3 as soon as at 4 days of life [19]. To investigate whether hAEC injection prevented complement activation, we measured circulating levels of C3 and analyzed C3 and C9 glomerular deposits. *Cfh*^*−/−*^ mice euthanized 10 days after hAEC injection exhibited significantly higher C3 serum levels compared to PBS-treated mice (Fig. 2A). Forty days after hAEC treatment, C3 levels were similar to those of PBS-treated mice. hAEC treatment induced a significant decrease in glomerular C3 deposits in *Cfh*^*−/−*^ mice analyzed 10 days post-hAEC injection, compared to PBS-treated mice (Fig. 2B, C). The score of C3 deposits in mice studied 40 days after cell injection was still lower than PBS-treated mice, but the difference did not reach statistical significance (Fig. 2B, C).

hAEC treatment was associated with a remarkable and significant reduction in glomerular deposits of C9 in mice 10 days after cell injection, compared to control mice (Fig. 2D, E), though this effect was lost 40 days after cell injection (Fig. 2D, E).

### hAEC ameliorated the glomerular ultrastructure of *Cfh*^*−/−*^ mice

**Fig. 3:**
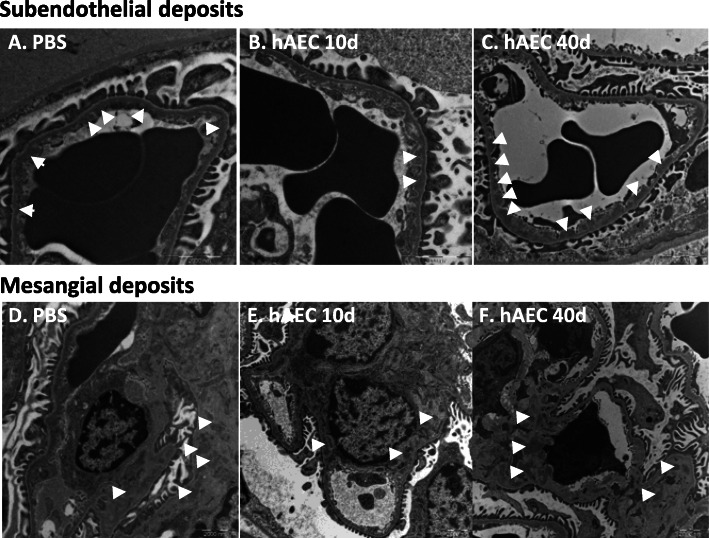
*hAEC reduced subendothelial and mesangial electron-dense deposits in Cfh*^*−/−*^ *mice.* Representative transmission electron micrographs showing subendothelial (**A**–**C**) and mesangial (**D**–**F**) electron-dense deposits (arrows) in glomeruli from *Cfh*^*−/−*^ *mice* given PBS (**A**, **D**) or given hAEC injection and analyzed 10 days (**B**, **E**) or 40 days (**C**, **F**) later

*Cfh*^*−/−*^ mice developed subendothelial electron-dense deposits at ultrastructural analysis at 2 months of age, culminating in urinary protein loss at 8 months of age [19]. We analyzed the glomerular structural integrity of kidneys from *Cfh*^*−/−*^ mice through transmission electron microscopy (TEM). Ultrastructural analysis showed the presence of subendothelial and mesangial electron-dense deposits in the glomeruli of PBS-treated *Cfh*^*−/−*^ mice (Fig. 3A, D). A reduction in subendothelial and mesangial deposits compared to PBS-treated mice was found in the glomeruli of mice analyzed 10 days post-hAEC injection (Fig. 3B, E). This effect was no longer detected at 40 days post-hAEC injection (Fig. 3C, F).

### hAEC induced an immune response

**Fig. 4:**
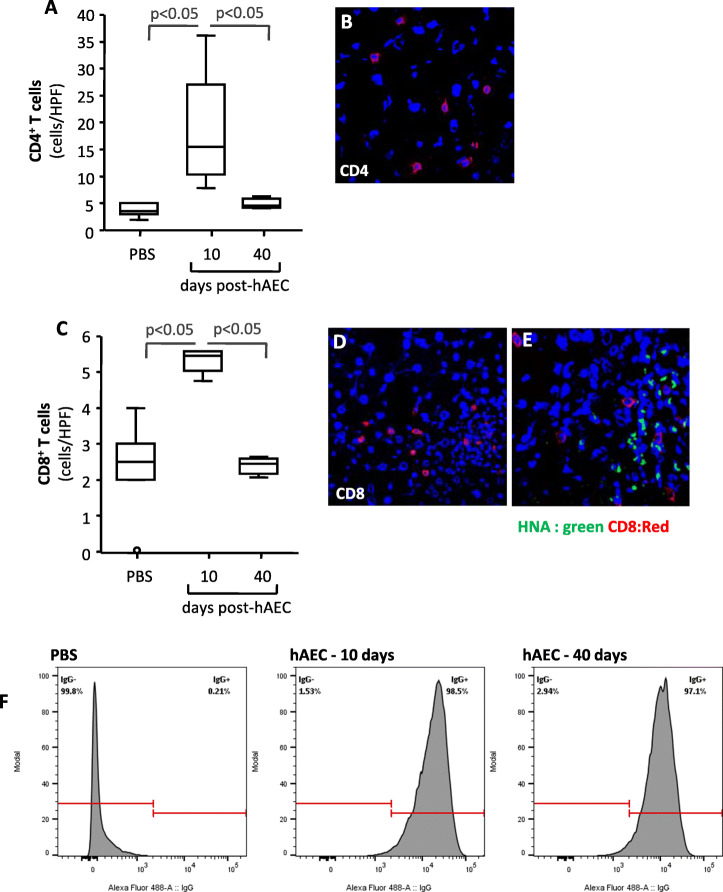
*hAEC induced cellular and antibody response.* Number expressed as cells/high power field (HPF) of liver infiltrating CD4^+^ T cells (**A**) and CD8^+^ T cells (C) in *Cfh*^*−/−*^ mice given PBS or analyzed either 10 or 40 days post-hAEC-injection. Horizontal lines indicate statistically significant differences (*P* < 0.05) between groups. Panels **B** and **D** are representative images of CD4^+^ or CD8^+^ T cells in livers from *Cfh*^*−/−*^ mice euthanized 10 days post-hAEC injection. Panel **E** provides a representative image of co-localization of CD8^+^ T cells (red) with HNA^+^ hAEC (green) 10 days post-hAEC injection in *Cfh*^*−/−*^ mice. Original magnification × 400. **F** FACS histograms for murine antibody binding to hAEC after exposing human cells to serum from PBS- or from hAEC-treated *Cfh*^*−/−*^ mice analyzed either 10 or 40 days after human cell injection

Based on the finding that hAEC engraftment was negligible 40 days after cell injection and the beneficial effects on markers of complement activation were almost lost at this time, we investigated whether human cells could be rejected in immunocompetent *Cfh*^*−/−*^ mice. To this end, we quantified CD4^+^ and CD8^+^ T cells in the livers of hAEC- or PBS-treated *Cfh*^*−/−*^ mice. Ten days after cell injection, a mild infiltrate of CD4^+^ and CD8^+^ T cells was found in the livers of *Cfh*^*−/−*^ mice (Fig. 4A–D), with the CD8^+^ T cells mainly found in the proximity of HNA^+^ hAEC clusters (Fig. 4E). The number of infiltrating CD4^+^ and CD8^+^ T cells in these mice was significantly higher than that found in the livers of PBS-treated mice (Fig. 4A–C). Forty days after hAEC injection, infiltrating CD4^+^ and CD8^+^ T cells were negligible, and their numbers were comparable to those found in PBS-treated *Cfh*^*−/−*^ mice (Fig. 4A–C). To investigate whether this mild T cell infiltration ultimately resulted in a humoral response, we measured murine antibodies against xenogeneic cells. At FACS analysis, we found that over 95% of hAEC stained positive for anti-mouse IgG after incubation with serum samples collected from mice euthanized either 10 or 40 days after hAEC injection, whereas a negligible number of hAEC stained positive for murine antibodies after incubation with serum from PBS-treated mice (Fig. 4F).

## Discussion

In this proof-of-principle study, we demonstrate that hAEC transplanted into the livers of newborn *Cfh*^*−/−*^ mice can supply FH, transiently preventing complement activation in the circulation and in renal glomeruli. hAEC attenuated C3 glomerular deposits and reduced C9 deposition. However, hAEC did not engraft long-term and the beneficial effects were ultimately lost, possibly due to hAEC rejection by immunocompetent *Cfh*^*−/−*^ mice.

hAEC are cells isolated from the human amniotic membrane and exhibit some of the features of stem cells, such as the ability to differentiate into multiple cells, including hepatocytes [13, 14]. There is experimental evidence that, following transplantation, undifferentiated hAEC engrafted in the liver and the hepatic microenvironment promoted their differentiation into hepatocyte-like cells expressing mature liver proteins and enzymes [20, 21] capable of ameliorating congenital metabolic liver diseases [14–16]. Liver-directed hAEC injection in newborn iMSUD mice increased liver BCKDH activity and corrected amino acid imbalance in the periphery and in the central nervous system, ultimately prolonging survival [15, 16]. In the Hurler Syndrome murine model, intrahepatic hAEC administration to newborn *Idua*^*−/−*^ mice restored the defective enzyme function, reduced systemic glycosaminoglycan accumulation, and improved disease phenotype [17].

In this study, we show that hAEC engrafted into the livers of *Cfh*^*−/−*^ mice after hepatic injections during the first 30 days of life and expressed human FH mRNA, although the levels of FH protein in serum were below the detection limit of the ELISA assay. However, finding that in hAEC-injected *Cfh*^*−/−*^ mice serum C3 levels were higher and glomerular C3 deposits were reduced compared to PBS-treated *Cfh*^*−/−*^ mice, indicates that hAEC engrafted into the livers were able to produce FH in sufficient quantities to partially prevent C3 activation and consumption in the circulation and to reduce the concomitant deposition of C3 activation fragments in glomeruli. These effects were comparable to those previously shown in *Cfh*^*−/−*^ mice receiving human plasma-derived FH and mini-FH molecules [10–12]. hAEC administration was also associated with reduced complement C9 in the glomeruli, indicating less activation of the terminal complement pathway. Most importantly, hAEC-induced correction of complement hyperactivation in the kidney translated into a substantial amelioration of glomerular ultrastructure, as illustrated by the reduction of the prototypical electron-dense deposits that were observed in vehicle-treated *Cfh*^*−/−*^ mice.

Earlier studies have shown that in *Cfh*^*−/−*^ mice the accumulation of C3 and C9 along the glomerular membrane was evident as soon as at 4 days of life [19]. The administration of hAEC early in life may have offered the advantage of providing FH—even at low levels—able to prevent the early phases of C3 and subsequently the C9 deposits, and to preserve the glomerular ultrastructure.

In *Cfh*^*−/−*^ mice, hAEC did not engraft long-term. hAEC were almost undetectable 40 days post-infusion, as were the expression of human FH and its effects on complement activation.

We followed the cell transplantation protocol found to be effective in iMSUD mice [15]. hAEC were given in the liver and in the early neonatal period, both conditions which have been shown to promote tolerance to human cells. Nevertheless, hAEC elicited an immune response and were eventually rejected, a finding that is in contrast with those observed in iMSUD mice [15, 16], in whom hAEC survived for 100 days. The longer duration of cell engraftment in iMSUD mice could be explained by the higher number of cells and by the repeated administrations—6 consecutive administrations for a total of 6 × 10^6^ cells—compared to two administrations of a more clinically applicable dose of a total of 2 × 10^6^ cells in our model. However, although hAEC are considered low-immunogenic and immunomodulatory cells [13], a mild infiltrate of T cells was found in the liver in proximity of hAEC and a strong antibody response against the xenogenic human cells developed in *Cfh*^*−/−*^ mice. The possibility exists that, in the context of an uncontrolled alternative pathway in *Cfh*^*−/−*^ mice, complement deposited on the xenogeneic hAEC surface, promoting both cell damage and opsonization and the consequent recognition by and activation of immune cells.

In conclusion, our data provide the first pieces of evidence that hAEC hold promise as a cellular strategy for enzyme replacement therapy in patients with genetic FH deficiency or abnormalities, who can benefit from combined liver/kidney transplantation. In the face of hAEC immunogenicity, this cell therapy may be applied to patients undergoing kidney transplantation in whom immunosuppression could protect hAEC from rejection, while hAEC may prevent disease recurrence, leaving the native liver and its physiological functions intact.

## Supplementary Information

**Additional file 1.** Supplemental information.

**Additional file 2: Supplemental Figure S1.** *Schematic overview of hAEC injections in Cfh*^*-/-*^ *mice.* hAEC or PBS were injected percutaneously into the livers of *Cfh*^-/-^ mice at 10 days of life (D.O.L.) and via the portal vein at 40 D.O.L. Mice were euthanized either 10 days or 40 days after the last hAEC injection (*n* = 3-5 mice per each group).

**Additional file 3: Supplemental Figure S2.** *Cfh*^*-/-*^ *given PBS as controls for hAEC injection or comparable markers of complement activation. Cfh*^*-/-*^ mice given PBS injection into the liver at 10 days of life and via the portal vein at 40 days of life, and euthanized either 10 days (PBS hAEC 10 days) or 40 days (PBS hAEC 40 days) after the last hAEC injection, exhibited comparable C3 serum levels (A), glomerular C3 (B) and C9 (C) deposits. Based on non-statistical differences in these parameters they were considered a single control group (P=NS).

**Additional file 4: Supplemental Figure S3.** *Phenotype of hAEC.* Representative FACS histograms for CD73 (A) and CD105 (B) expression and dot plots for HLA-ABC and HLA-DR expression (C) f hAEC.

## Data Availability

The dataset used and analyzed during the current study are available from the corresponding author upon reasonable request.

## Abbreviations

aHUS: Atypical hemolytic uremic syndrome
BCKDH: Branched chain α-keto acid dehydrogenase
C3G: C3 glomerulopathy
Cfh: Murine complement factor h gene
ELISA: Enzyme linked immunosorbent assay
FH: Human factor H
hAEC: Human Amnion Epithelial Cells
HNA: Human nuclear antigen
IDUA: α-L-Iduronidase
iMSUD: Intermediate Maple Syrup Urine Disease
PBS: Phosphate-buffered saline
TEM: Transmission electron microscopy
α1AT: α1 Anti-trypsin
