# Supplementary material for: Amnion epithelial cells are an effective source of factor H and prevent kidney complement deposition in factor H-deficient mice
Source: Stem Cell Res Ther. 2021 Jun 10;12:332. doi: 10.1186/s13287-021-02386-7 (PMC8194190; doi:10.1186/s13287-021-02386-7)
Supplement: Supplementary file 1 — Additional file 1. Supplemental information. [file 13287_2021_2386_MOESM1_ESM.docx]

**Amnion Epithelial cells are an effective cell source of factor H and prevent kidney complement deposition in factor H-deficient mice**

**By Federica Casiraghi et al.**

**Supplemental information**

*Mice*

*Cfh^–/–^* mice^1^ were kind gifts from Dr Matthew Pickering at Imperial College in London and a colony was maintained in the Envigo animal care facility through littermate crossing.

Procedures involving animals and their care conformed with institutional guidelines in compliance with national (D.lgs 26/2014; Authorization n.19/2008-A issued March 6, 2008 by Ministry of Health); the NIH Guide for the Care and Use of Laboratory Animals (2011 edition) and EU directives and guidelines (EEC Council Directive 2010/63/UE).

All animal experimental protocols were approved by our Institutional Committee (IACUC, IRFMN Animal Care and Use Committee) at the Istituto di Ricerche Farmacologiche Mario Negri IRCCS, which includes “ad hoc” members for ethical issues. Animals were housed in the Institute’s Animal Care facilities, which meet international standards.

PBS-treated mice as controls for hAEC injections exhibited non-significant differences in all the considered parameters (Supplemental Figure S2) and were therefore pooled in a single control group (n=6).

*Isolation and phenotype of hAEC*

hAEC were isolated from human amniotic membrane as previously described^2^ at the Department of Laboratory Medicine, Karolinska Institute. Isolated hAEC were suspended in complete media supplemented with 10% DMSO and cryopreserved in vials in liquid nitrogen and shipped to the Istituto di Ricerche Farmacologiche Mario Negri IRCCS according to standard procedures.

Aliquots of thawed hAEC were incubated for 30 minutes at 4°C with the following antibodies: PE-conjugated mouse anti-human CD73 (clone AD2, BD), PE-conjugated mouse anti-human CD105 (clone SN6, Thermofisher), PE-conjugated mouse anti-human HLA-ABC (clone G46-2.6, BD), BV605-conjugated anti HLA-DR (clone G46-6, BD). Cells were then acquired by FACS Fortessa X-20 (BD Bioscience) and analyzed with FlowJo software. Trypan blue exclusion showed a hAEC viability >85% prior to injection.

Before injection, undifferentiated hAEC showed high expression of CD73, low expression of CD105, were negative for the HLA-DR and positive for HLA-ABC (Supplemental Figure S3), consistent with the previously reported hAEC phenotype.

*Liver and kidney immunohistochemistry*

Mouse liver and kidney sections (3µm) were cut with a cryostat, air-dried, and fixed with acetone and analyzed using immunofluorescence techniques on frozen tissue sections.

To assess liver hAEC engraftment, liver sections were incubated with anti-hepatocyte antigen antibody [OCH1E5] followed by Cy3 conjugated donkey anti-mouse IgG and then with AF488-conjugated mouse anti-Human Nuclear Antigen (HNA) antibody (Millipore, clone: 235-1). Cell nuclei were counterstained with DAPI. At least 10 HPF were analyzed and percentages of HNA^+^ cells on DAPI^+^ cells were calculated for each animal.

Kidneys were analyzed for complement C3 and C9 deposition. Frozen kidney sections were incubated with FITC-conjugated goat anti-mouse C3 (polyclonal, Cappel) or rabbit anti-mouse C9 (kindly provided by Dr. Daha, Department of Nephrology, Leiden University Medical Center, The Netherlands) followed by Cy3-conjugated goat anti-rabbit (Jackson Immunoresearch Laboratories). C3 or C9 deposition was scored (0=absent; 1=faint staining; 2=moderate staining; 3=intense staining). Around 10 glomeruli for each section were examined. Infiltrating T cells were analyzed on frozen hepatic sections using an immunofluorescence technique with primary rat anti-mouse CD4 (clone RM4-5, Biolegend) or rat anti-mouse CD8a (clone 53-6.7, BioLegend) antibodies, both followed by secondary goat anti-rat Cy3 antibody (Jackson). Negative controls were run parallel by omitting the primary antibody. CD4^+^ and CD8^+^ T cells were counted in at least 10-15 randomly selected HPF (X400).

*Measurement of C3 and FH levels in serum*

Circulating C3 levels were quantified in serum using kit Mouse Complement Factor 3 (Genway Biotech Inc). The ELISA assay was performed according to the manufacturer’s instructions.

FH levels in serum samples were assessed by ELISA, as previously described.^3^

*Ultrastructural analysis*

Fragments of kidney tissue were fixed overnight in 2.5% glutaraldehyde (Sigma-Aldrich, St. Louis, MO) in 0.1 M sodium cacodylate buffer (pH 7.4) (Electron Microscopy Sciences, Hatfield, PA, USA) and washed repeatedly in the same buffer. After postfixation in 1% OsO_4_, specimens were dehydrated through ascending grades of alcohol and embedded in Epon resin. Ultrathin sections were stained with UranyLess (Electron Microscopy Sciences, Hatfield, PA, USA) and lead citrate (Electron Microscopy Sciences) and examined using transmission electron microscopy (TEM, Morgagni 268D, Philips, Brno, Czech Republic). Three glomeruli per animal were analyzed.

*PCR analysis*

Liver sections were snap-frozen in liquid nitrogen and stored at -80°C. Total RNA was extracted using the NucleoSpin RNA kit (Macherey-Nagel) according to the manufacturer’s protocol. RNA was reverse transcribed to cDNA using Superscript II (Invitrogen). Quantitative real-time PCR was conducted on the ABI PRISM 7300 Real Time-PCR System (PE Applied Biosystems) with Power SYBER Green Master Mix and the following specific primers: human FH, forward: 5’-cctccatcatgcataaaaaca-3’, reverse: 5’-tgttacattactggctccatcc-3’; human α1 anti-tripsin (α1AT), forward: 5’-ctgaatttcaacctcacggagat-3’, reverse: 5’-ggttgagggtacggaggagtt-3’. Mouse β2-microglobulin, forward: 5’-aagtgggatcgagacatgtaagc-3’, reverse: 5’-tcatccaatccaaatgcgg-3’ and mouse gapdh, forward: 5’-TCATCCCTGCATCCACTGGT-3’; reverse: 5’- CTGGGATGACCTTGCCCAC-3’ were included as housekeeping. The ∆∆ threshold cycle technique was used to calculate cDNA content in each sample using the cDNA expression in a mouse given hAEC and euthanized 40 days later as reference (calibrator). Melting curve analysis showed a single dissociation peak for all PCR gene products, confirming the specificity of the reactions. No amplification was found in control reactions without cDNA.

*Determination of serum levels of anti-hAEC antibodies*

hAEC (0.25 x 10^6^ cells) were incubated with murine serum samples diluted 1:100, washed and then incubated with rabbit anti-mouse Alexa-Fluor 488 secondary antibody. As negative controls, hAEC were incubated either with PBS or with serum from non-injected C57 wild type mice (>99% IgG negative cells).

*Statistical analysis*

Data are represented as box and whisker plot with outliers. The significance of the differences between individual groups was analyzed by one-way ANOVA. All data were analyzed using MedCalc 10.0.1 statistical software. Differences with a P value <0.05 were considered significant.

*References*

1. Pickering MC, Cook HT, Warren J, et al. Uncontrolled C3 activation causes membranoproliferative glomerulonephritis in mice deficient in complement factor H. *Nat Genet*. 2002;31(4):424-428. doi:10.1038/ng912

2. Gramignoli R, Srinivasan RC, Kannisto K, Strom SC. Isolation of Human Amnion Epithelial Cells According to Current Good Manufacturing Procedures. *Curr Protoc Stem Cell Biol*. 2016;37:1E.10.1-1E.10.13. doi:10.1002/cpsc.2

3. Valoti E, Alberti M, Iatropoulos P, et al. Rare Functional Variants in Complement Genes and Anti-FH Autoantibodies-Associated aHUS. *Front Immunol*. 2019;10:853. doi:10.3389/fimmu.2019.00853
