# Supplementary material for: Amnion epithelial cells are an effective source of factor H and prevent kidney complement deposition in factor H-deficient mice
Source: Stem Cell Res Ther. 2021 Jun 10;12:332. doi: 10.1186/s13287-021-02386-7 (PMC8194190; doi:10.1186/s13287-021-02386-7)
Supplement: Supplementary file 2 — Additional file 2: Supplemental Figure S1. Schematic overview of hAEC injections in Cfh-/- mice. hAEC or PBS were injected percutaneously into the livers of Cfh-/- mice at 10 days of life (D.O.L.) and via the portal vein at 40 D.O.L. Mice were euthanized either 10 days or 40 days after the last hAEC injection (n = 3-5 mice per each group). [file 13287_2021_2386_MOESM2_ESM.pdf]

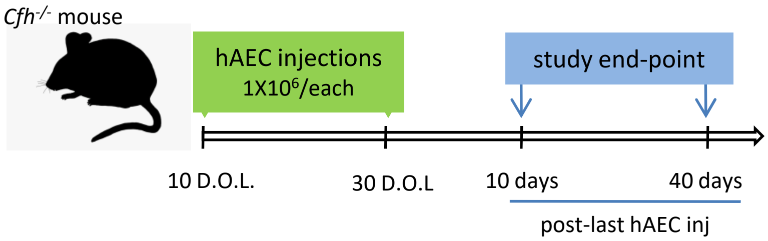

Supplementary Figure S1. Schematic overview of hAEC injections in Cfh$^{-/-}$ mice. hAEC or PBS were injected percutaneously in the liver of *Cfh*$^{-/-}$ mice at 10 days of life (D.O.L.) and via portal vein at 40 D.O.L. Mice were euthanized either 10 days or 40 days after the last hAEC injection (n=3-5 mice per each group).

Supplementary Figure S1
