# Supplementary material for: Amnion epithelial cells are an effective source of factor H and prevent kidney complement deposition in factor H-deficient mice
Source: Stem Cell Res Ther. 2021 Jun 10;12:332. doi: 10.1186/s13287-021-02386-7 (PMC8194190; doi:10.1186/s13287-021-02386-7)
Supplement: Supplementary file 3 — Additional file 3: Supplemental Figure S2. Cfh-/- given PBS as controls for hAEC injection or comparable markers of complement activation. Cfh-/- mice given PBS injection into the liver at 10 days of life and via the portal vein at 40 days of life, and euthanized either 10 days (PBS hAEC 10 days) or 40 days (PBS hAEC 40 days) after the last hAEC injection, exhibited comparable C3 serum levels (A), glomerular C3 (B) and C9 (C) deposits. Based on non-statistical differences in these parameters they were considered a single control group (P=NS). [file 13287_2021_2386_MOESM3_ESM.pdf]

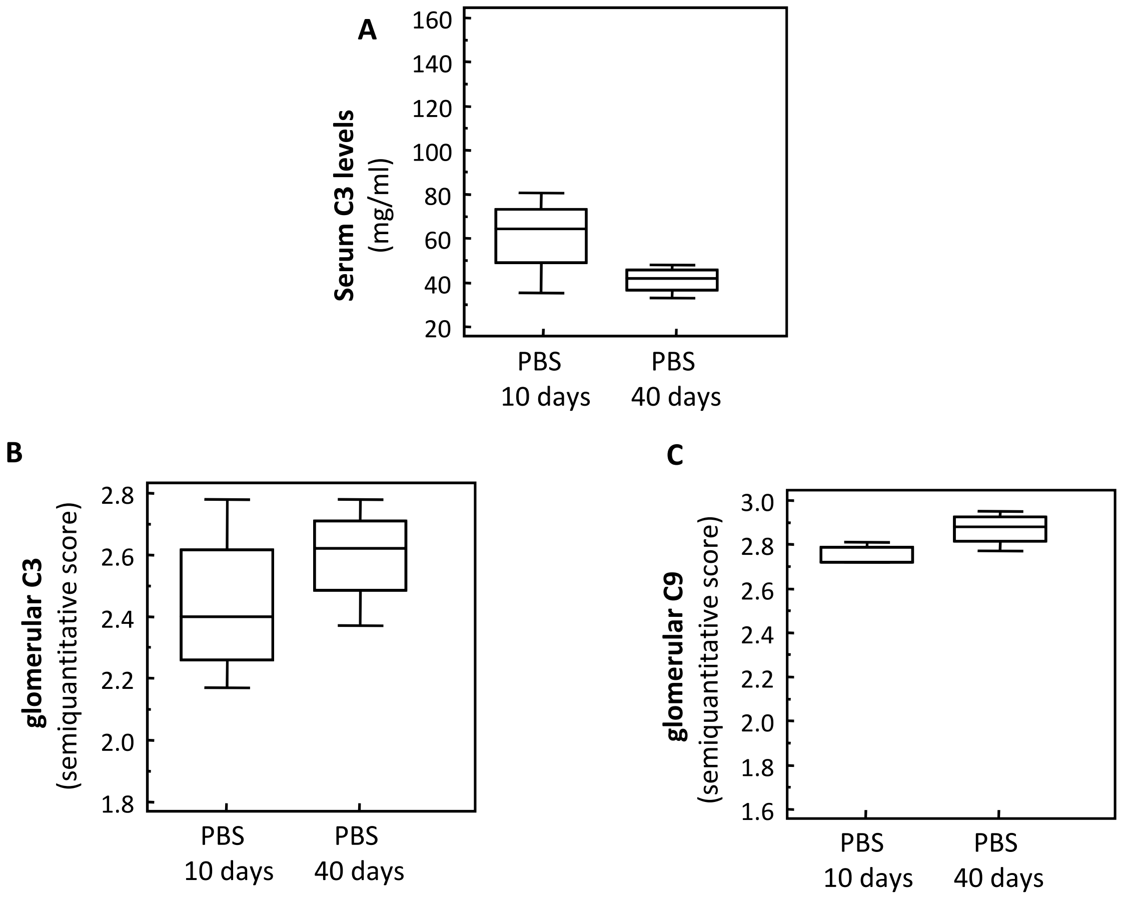

Supplementary Figure S2. *Cfh$^{-/-}$* given PBS as controls for hAEC inejction and analysed at either 10 or 40 days after showed comparable markers of complement activation. *Cfh$^{-/-}$* mice given PBS injection in the liver at 10 days of life and via portal vein at 40 days of life and euthanized either 10 days (PBS hAEC 10 days) or 40 days (PBS hAEC 40 days) after the last hAEC injection showed comparable C3 serum levels **(A)**, glomerular C3 **(B)** and C9 **(C)** deposits. Based on the non statistical differences in these parameters they were considered as a single control group (P=NS).
