# Supplementary material for: Amnion epithelial cells are an effective source of factor H and prevent kidney complement deposition in factor H-deficient mice
Source: Stem Cell Res Ther. 2021 Jun 10;12:332. doi: 10.1186/s13287-021-02386-7 (PMC8194190; doi:10.1186/s13287-021-02386-7)
Supplement: Supplementary file 4 — Additional file 4: Supplemental Figure S3. Phenotype of hAEC. Representative FACS histograms for CD73 (A) and CD105 (B) expression and dot plots for HLA-ABC and HLA-DR expression (C) f hAEC. [file 13287_2021_2386_MOESM4_ESM.pdf]

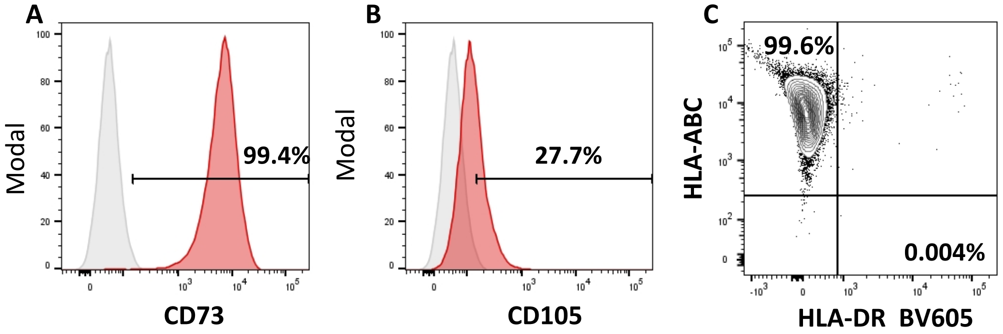

Supplementary Figure S3. Representative FACS histograms for CD73 (A) and CD105 (B) expression and dot plots for HLA-ABC and HLA-DR expression (C) of hAEC.
